# Addressing inequities in nutritional supplementation in tuberculosis: implementation research from Puducherry, India

**DOI:** 10.5588/pha.26.0021

**Published:** 2026-08-24

**Authors:** R. Gouroumourty, K.M. Akshaya, M. Verma, P. Kandasamy, A.R. Dongre, S. Mathivanan, N. Sabarish, M. Natarajan, M.M. Regy, V. Couppoussamy

**Affiliations:** 1 Department of Community Medicine, Sri Manakula Vinayagar Medical College and Hospital, Puducherry, India;; 2 Department of Community Medicine, Yenepoya Medical College, Yenepoya (Deemed to be University), Mangaluru, India;; 3 Department of Community and Family Medicine, All India Institute of Medical Sciences, Bathinda, India;; 4 Department of Pulmonary Medicine, Madras Medical College, Chennai, India;; 5 World Health Organization, NTEP Technical Support Network, Bangalore, India;; 6 State TB Cell, Government of Puducherry, Puducherry, India.

**Keywords:** tuberculosis, nutritional support, underweight, poverty, M-PHISOR

## Abstract

**SETTINGS:**

Operational research conducted in Puducherry identified gaps in achieving an equitable distribution of food baskets to address undernutrition among all patients with tuberculosis (TB) and drug-resistant TB (DR-TB).

**OBJECTIVE:**

To assess the changes in food basket distribution following the implementation of a prioritisation mechanism in Puducherry between June and August 2025, as compared to June and August 2023. This cohort study used secondary programme data to compare outcomes before and after implementation.

**RESULTS:**

In 2025 (N = 73), the time to receive the first food basket from diagnosis was reduced by 39% (*P* = 0.003) in all with undernutrition and below poverty line, compared with 2023 (N = 53). Similarly, the number of food baskets they received increased by 30% (*P* = 0.048). The time to receive the first food basket from diagnosis and the number of food baskets improved for patients with DR-TB.

**CONCLUSION:**

Patients with TB with undernutrition and below the poverty line received food baskets earlier in 2025 than in 2023. Attempts to prioritise the number of food baskets to these vulnerable groups also improved modestly. Real-time monitoring of indicators for a wider cohort and strengthening community engagement will help sustain the distribution in the long run.

Undernutrition and tuberculosis (TB) share a bidirectional relationship, with more than one third of all TB cases attributable to undernutrition in India.^1^ Notably, those with severe undernutrition were at a fourfold increased risk of mortality.^2^ Poverty is an important social determinant of TB and is closely linked with the development of undernutrition.^3^ Estimates from the survey conducted between 2018 and 2023 reveal that 22.9% of the Indian population is multidimensionally poor.^4^ Studies have shown that impoverished individuals were unable to gain weight during the course of TB treatment despite being effective.^5,6^ The nutritional support initiatives under the National Tuberculosis Elimination Programme in India include Nikshay Poshan Yojana, which provides a monthly direct benefit cash transfer of 1000 INR (∼12 USD).^7^ Another initiative, *Ni-Kshay* (End TB) *Mitra* (Friend), was launched in September 2022. ‘Ni-Kshay Mitras’ are community-based individuals, NGOs, Cooperative Societies, elected representatives, and corporates who volunteer to ensure the provision of monthly nutritional kits to patients with TB (PwTB).^8^ Studies about the *Ni-Kshay Mitra* initiative in India broadly discuss evaluation, implementation challenges, and drivers of community participation in various settings.^9–12^ In line with this, operational research was conducted in Puducherry to assess coverage, delivery models, and implementation challenges, adding to the evidence base to guide practice and policy.^13^

It was found that the majority of PwTB in Puducherry were below poverty line (BPL). Key outcomes, such as the number of food baskets received and the time to receipt of the first food basket after diagnosis, did not differ by nutritional status.^13^ The reasons being: fewer donors, shortage of supplies, and variations in distribution across different health facilities.^13^ There is a scarcity of evidence on the targeted prioritisation of food baskets in a donor-dependent system. Hence, we aimed to assess changes in food basket distribution under this initiative following the implementation of a prioritisation mechanism for the vulnerable populations, such as BPL PwTB with undernutrition (body mass index [BMI] < 18.5) and drug-resistant TB (DR-TB) in Puducherry.

## METHODS

This cohort study used secondary programme data of PwTB collected across two time periods: June–August 2023 and June–August 2025. The exposure included those who consented to nutritional support, and the outcome was the number of food baskets received and the time to receive the first food basket. This study was conducted between June 2025 and February 2026.

### Study setting

The study was conducted in the Puducherry district of the Union Territory of Puducherry, with a population of 15.82 lakhs as of 2023.^14^ An operational research study conducted in Puducherry between 2024 and 2025 details the study setting, the food basket delivery model in Puducherry district, and the characteristics of *Ni-Kshay Mitras*.^13^

### Study population

All the adult PwTB, including those with DR-TB initiated on treatment from Peripheral Health Institutions (PHIs) and consented to nutritional support, were included.

### Theory of change

**FIGURE 1.**
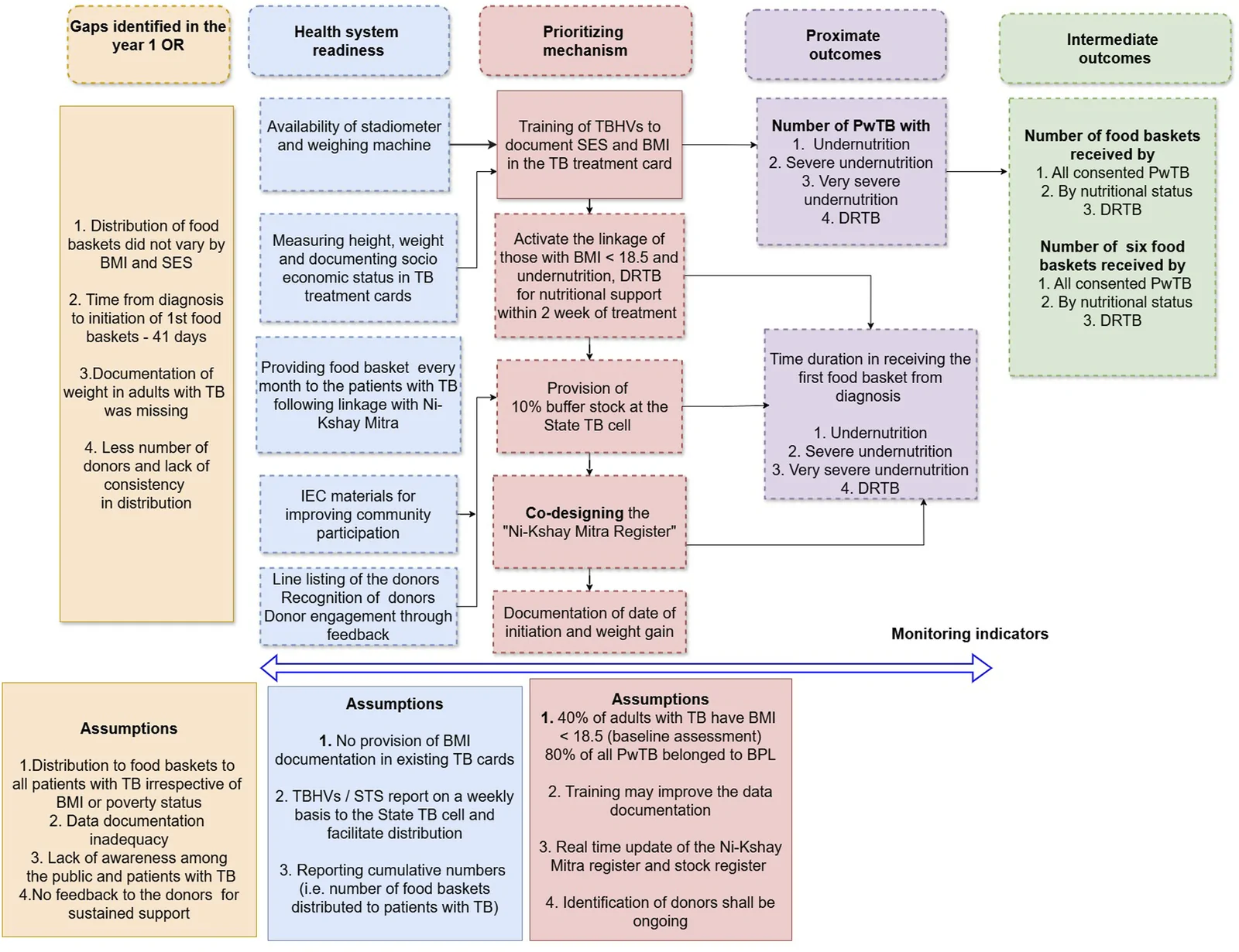
Theory of change – co-designed implementation research on food basket delivery among vulnerable populations with TB in Puducherry, India. TB = tuberculosis; BMI = body mass index; IEC = information, education, and communication material; OR = operational research; TBHVs = TB health visitors; STS = Senior Treatment Supervisor; SES = socio-economic status; PwTB = patients with TB; DRTB = drug-resistant TB.

The theory of change (TOC) is based on the guidance provided by the updated Medical Research Council framework to develop appropriate methods to implement and evaluate complex interventions.^15^ The prioritisation mechanism for food basket delivery to the vulnerable population with TB was based on interventions that worked in the routine programmatic setting, as well as barriers and suggestions from stakeholders’ perspectives (Figure 1). The mechanism focused on adding three components to the existing programmatic setting, with assumptions based on our baseline findings (Supplementary Data Table S1). The first component included initial training for all TB health visitors (TBHVs) and Senior Treatment Supervisors (STSs) from the 34 PHIs, as well as the pharmacist at the state TB cell in Puducherry. The training included calculating and documenting BMI and socio-economic status, and monthly weight on the treatment cards and in the co-designed register. The criteria for prioritisation were to distribute food baskets within two weeks or at the earliest, among those with a BMI <18.5 and BPL, and DR-TB. Following the distribution of food baskets, the distribution date for each basket, up to the end of treatment, and the corresponding weight were recorded. From this, the key outcomes were derived. The second component included maintaining a 10% reserve of food baskets at the State TB cell level. This step was undertaken to ensure a minimum stock of food baskets when the donor supply was expected to be reduced. The stock maintenance including the distribution to the PHIs and documentation was done by pharmacist at the State TB cell. The performance of prioritisation was monitored using the indicators, and the feedback was provided to the stakeholders (Supplementary Data Table S2).

### Data collection

Secondary data on the socio-demographic, diagnostic, and treatment characteristics of the PwTB were obtained from TB treatment cards and entered using EpiCollect 5. The date of distribution of the first food basket from the date of diagnosis, and the total number of food baskets, were obtained from the *Ni-Kshay Mitra* register (PHIs) and entered into Excel. *Ni-Kshay’s* patient ID was used to compile information from two sources. The information for the June–August 2023 cohort was extracted from the overall data (April 2023 to March 2024) of our study conducted in Puducherry during 2024.^13^ In case of any discrepancy or incompleteness, the data were verified against the treatment cards and registers, and duplicate entries were removed. Operational definitions were the same as those used in our previous study.^13^

### Data analysis

The data analysis was done using R software (version 4.4.1). The continuous variables were summarised as median (interquartile range [IQR]) following the Shapiro–Wilk test of normality, and the categorical variables were summarised as number (%). An equity-sensitive improvement was operationally defined as a shorter time to receiving the first food basket and a higher number of food baskets, based on BPL with undernutrition and DR-TB statuses. The changes in the key outcomes were summarised as effect sizes using the rank biserial coefficient (non-parametric), and the Mann–Whitney U test was used to determine significance. A multivariate negative binomial mixed model regression (skewed discrete data with unequal mean and variance) was applied to determine whether individuals with undernutrition and BPL in the June–August 2025 cohort received food baskets earlier and in greater numbers than in 2023, after adjusting for confounders. The independent fixed-effect variables (cohort, age, sex, occupation, number of household contacts, and TB type) were chosen a priori for programmatic relevance, as they influence food basket distribution after accounting for PHI (random effects). A *P* value less than 0.05 was considered significant. An available case analysis approach was used to account for missing data. The sensitivity analysis was conducted with and without missing data, and the latter was presented.

### Ethical statement

Approval from the institutional ethics committee of the ICMR National Institute of Epidemiology, India (NIE/IHEC/A/202506-20 dated 29/6/2025) was obtained. A waiver of written informed consent was obtained for the use of secondary data.

## RESULTS

Of the total 296 PwTB notified between June and August 2025, 216 were initiated on treatment from the PHIs. Similarly, 207 PwTB were initiated on treatment during 2023 (Supplementary Data Table S3).

### Implementation fidelity indicators

All 13 TBHVs and 5 STSs in Puducherry were sensitised and trained on the prioritisation mechanism and documentation during June and July 2025. All 34 PHIs reported BMI on the TB treatment cards and documented monthly nutrition support and PwTB’s weight in the co-designed *Ni-Kshay Mitra* register. Out of 9 months from June 2025 to Feb 2026, maintaining a 10% buffer stock was feasible for 4 months.

### Primary proximate outcomes

**FIGURE 2.**
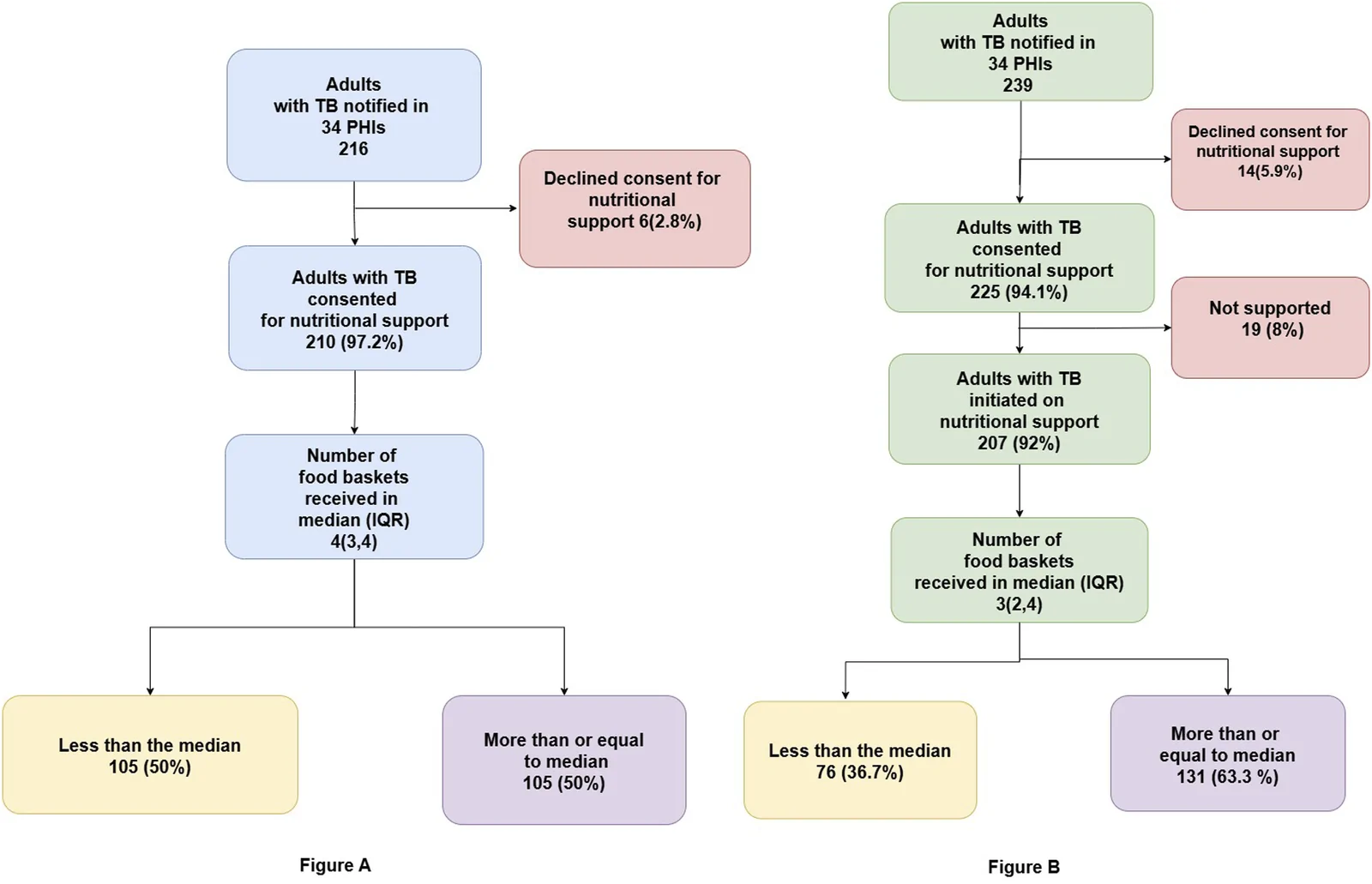
Implementation cascade of nutritional support to the adults with TB notified from peripheral health institutions (PHIs) in Puducherry district, India, between June–August 2025 and June–August 2023. TB = tuberculosis; IQR = interquartile range.

Of the 210 PwTB who consented to nutritional support in 2025, the median (IQR) number of food baskets received was 4 (3,4), and 105 (50%) received at least 4 food baskets during treatment, as compared to 2023 (Figure 2). Approximately 45 (21.4%) of PwTB received nutritional support within 2 weeks of diagnosis. About 13 (5.7%) and 11 (7.9%) PwTB received six food baskets during their course of treatment in the 2025 and 2023 cohorts, respectively.

### Equity analysis

Overall, the time to receipt of the first food basket from diagnosis was 28 (17,44) and 31 (21,48) days for all PwTB in 2025 and 2023, respectively. There was a statistically significant difference in the time to receive the first food basket from diagnosis between BPL individuals with undernutrition and those with severe undernutrition, with the June–August 2025 cohort demonstrating higher ranks than the 2023 cohort (Table 1).

**TABLE 1.** Differences in distribution of food baskets to patients with tuberculosis (TB) belonging to below poverty line notified from peripheral health institutions during June–August 2023 and June–August 2025 in Puducherry district, India, by undernutrition and drug-resistant TB.

	June–August 2023^A^			June–August 2025			Effect size^B^	
a) Time duration (in days) for receiving the first food basket								
Levels of undernutrition	N	Median	IQR	N	Median	IQR		*P* value^C^
Undernutrition (not severe)	33	49	35,73	40	31	23,46	0.409	0.003
Severe undernutrition	12	48	23,63	26	27	17,39	0.349	0.090
Very severe undernutrition	8	18	15,54	7	16	1,45	0.161	0.643
b) Number of food baskets received by adults with TB								
Levels of undernutrition	N	Median	IQR	N	Median	IQR		*P* value
Undernutrition (not severe)	33	3	2,4	40	4	3,4	−0.323	0.015
Severe undernutrition	12	2	2,3	26	4	3,4	−0.474	0.019
Very severe undernutrition	8	4	3,4	7	5	2,6	−0.411	0.184
c) Drug-resistant TB								
	N	Median	IQR	N	Median	IQR		*P* value
Time duration for receiving the first food basket	7	95	29,170	13	22	12,36	0.505	0.074
Number of food baskets received by adults with TB	7	3	2,5	13	5	4,5	−0.374	0.182
Number of six food baskets received by adults with TB^D^	7	1	14.3	13	3	23.7	–	–

IQR = interquartile range.

A Two data related to body mass index were missing.

B Rank biserial correlation.

C Mann–Whitney U test.

D Row percentage.

### Sub-group analysis among those with undernutrition and BPL

About 53 individuals from the June–August 2023 cohort and 74 individuals from the June–August 2025 cohort who were in the undernutrition category and BPL were included in the regression (Table 2). Two models were run for each dependent outcome based on available data. Vulnerable PwTB in the June–August 2025 cohort experienced a statistically significant 39% reduction in time to receive their first food baskets compared to the baseline cohort (*P* = 0.003) – see Table 2. The number of food baskets received by PwTB in the June–August 2025 cohort was 30% higher than in the baseline cohort, although this difference was not significant (*P* = 0.048).

**TABLE 2.** Socio-demographic and clinical predictors of diagnosis to the receipt of first food basket and total number of food baskets received among those patients with tuberculosis (TB) with undernutrition and below poverty line status, Puducherry district, India.

	Diagnosis to the receipt of the first food basket			Number of food baskets received		
Characteristic	Exp (Beta)	95% CI	*P* value	Exp (Beta)	95% CI	*P* value
Cohorts						
June–August 2023	–	–		–	–	
June–August 2025	0.61	0.47, 0.85	**0.003** ^A^	1.30	1.00, 1.69	**0.048** ^A^
Age (years)	1.00	0.99, 1.02	0.5	1.00	0.99, 1.00	0.4
Sex						
Male	–	–		–	–	
Female	0.87	0.55, 1.37	0.6	1.14	0.82, 1.58	0.4
Occupational status						
Working	–	–		–	–	
Not working	1.14	0.76, 1.74	0.5	1.03	0.76, 1.41	0.8
Type of TB						
Clinically diagnosed	–	–		–	–	
Bacteriologically confirmed	0.75	0.43, 1.29	0.3	1.20	0.80, 1.81	0.4
Number of household contacts	1.06	0.94, 1.19	0.4	0.95	0.86, 1.05	0.3

CI = confidence interval.

A Statistically significant for the outcome, diagnosis to the receipt of the first food basket, while it was nearly significant for the outcome, number of food baskets received after adjusting for the confounders.

### Secondary outcome – weight status of PwTB by nutritional statuses

Of the 210 PwTB initiated on nutritional support in the June–August 2025 cohort, weight (kg) at the end of the intensive phase was available for 197 (93.8%), whereas in the June–August 2023 cohort (N = 207), it was available for 97 (46.8%). The difference in the end intensive phase weight (kg) of PwTB from the baseline weight was documented as no change (92, 46.7%), weight gain (93, 47.2%), or weight loss (12, 6.1%). Of the 93 PwTB who documented an increase in weight, 13 (14%) had undernutrition, 16 (17.2%) had severe undernutrition, and 4 (4.3%) had very severe undernutrition.

## DISCUSSION

The findings of our study were based on the key recommendations from our baseline study to strengthen the *Ni-Kshay Mitra* initiative in Puducherry.^13^ TOC was used to plan the prioritisation of food basket distribution for PwTB in our study. Unlike TB, the TOC framework was adopted in planning interventions for people with HIV and as a roadmap for universal health coverage.^16,17^ Following the implementation of prioritisation in 2025, we found that the time to receive the first food basket decreased significantly compared to 2023.

The prioritisation was widely adopted by all the TBHVs and STS in Puducherry, as indicated by lower missing data on BMI and socio-economic status. Initially, the reporting format was paper-based; later, it was incorporated into a co-designed register-based format. One in five PwTB received the first food basket within 2 weeks of treatment initiation. Possible reasons include donor availability and the TBHVs’ motivation to establish early nutritional support, which was part of our TOC assumption. The availability of donors enabled maintaining a 10% buffer stock, which was feasible for four of the nine months.

We found that the median time to receiving the first food basket from diagnosis was reduced by 39% among PwTB with undernutrition belonging to BPL. This may be explained by two reasons: attempts to maintain reserve stock and the initiation of nutritional support as soon as the patient is started on treatment at the PHIs. The latter is emphasised by measuring the BMI and strengthening the nutritional support amongst those in the vulnerable group. The median number of food baskets received by PwTB in 2025 increased by 30% from 2023. Although not statistically significant, it was programmatically relevant, given the donor-dependent context. Also, clinically, nutritional support will help in combating side effects associated with anti-tubercular drug therapy. Not much difference was observed in the key outcomes among those with very severe undernutrition, as current practices in Puducherry include the admission of patients with very severe undernutrition, followed by inpatient care for one month, which was variable.

Documenting end intensive phase weight (kg) may serve as a proxy for improved treatment outcomes.^18^ The TOC was instrumental in motivating the TBHVs/STS to document weight, as reflected in the data availability in 2025 compared to 2023. In our study, weight gain was documented in less than half of the PwTB in the 2025 cohort. Interpreting weight gain must be done cautiously due to the large number of missing data in 2023 and limitations in data validation, and it cannot be causally linked to nutritional supplementation in a routine programmatic setting. We need to refine the prioritisation criteria further to include the existing policy on the admission of PwTB with severe and very severe undernutrition in Puducherry. Also, efforts should be explicitly defined on when and how to initiate nutritional support for these PwTB at the earliest. Routine monitoring by STS and the medical officers using the proposed indicators may aid in planning nutritional support for their PHIs. The sustainability of prioritisation depends on donor availability and thus calls for active participation of *Ni-Kshay Mitras* through an intensified media campaign and donor identification by the state TB cell. This paves the way forward for context-specific community engagement through participatory action research. Further, research should focus on the long-term outcomes of prioritisation, including maintenance and adoption.

Prioritisation of nutritional supplementation was implemented in the context of community-based management of severe and moderate acute malnutrition for children under five in India.^19^ Our study is among the first few to prioritise nutritional supplementation in a routine programmatic setting for PwTB, utilising existing resources. The real-time data on the number of food baskets received and the actual consumption by the PwTB could not be verified. Measurement bias in BMI assessment and missing data add to the study limitations.

## CONCLUSION

The implemented prioritisation mechanism resulted in a reduction in the time to initiation of the first food basket from diagnosis to BPL PwTB with undernutrition and DR-TB in 2025. The number of food baskets they received modestly improved compared to 2023. Sustaining prioritisation efforts requires actively mobilising donors by fostering community engagement and by regularly monitoring progress through indicators. Future efforts should aim to reduce the time to the initiation of food baskets among those with severe and very severe undernutrition.

## Supplementary Material
