# Supplementary material for: Addressing inequities in nutritional supplementation in tuberculosis: implementation research from Puducherry, India
Source: Public Health Action. 2026 Aug 24;16(3):150–5. doi: 10.5588/pha.26.0021 (PMC13502498; doi:10.5588/pha.26.0021)
Supplement: Supplementary file 1 [file pha26-0021_supplementarydata1.pdf]

Supplementary Table 1: Components in the theory of change of the co-designed implementation research on food basket delivery among vulnerable populations with Tuberculosis in Puducherry, India

Components	Description	Assumptions
**Component 1**<br>Document the BMI and SES, followed by identification and linkage of those with undernutrition and below poverty status.	Training of TBHVs to document BMI and socio-economic statuses in the TB treatment card.	TBHVs were trained to document weight and height and to compute BMI.<br><br>A stadiometer and a weighing machine were available in all the PHIs.<br><br>However, there was no provision of BMI documentation in the TB treatment card.<br><br>Also, the data documentation of socio-economic status was incomplete, based on the baseline OR findings.
	Documentation will enable identification as soon as the patient with TB approaches the current PHI for treatment.	Our baseline findings showed that food baskets were distributed to all the PwTB, irrespective of their vulnerability.<br>Also, the time to the receipt of the first food basket was 41 days from the treatment initiation.
	Linkage for nutritional support by the TBHV through communication to the STS within two weeks.	Also, the communication is done at the end of every month to disburse food baskets in the 1st week of the upcoming month.
**Component 2**<br>Maintaining the reserve of food baskets at the State TB cell for early initiation	Ensuring buffer stock maintenance (10% of the total supplies) at the State TB cell. This was planned based on the large-scale food baskets received from NGOs/ Corporate/ Educational institutions.	Based on our year 1 findings, the State TB cell stock was maintained by the pharmacist using the stock register.<br><br>No provision of buffer stock.<br><br>This measure aimed at enabling the TBHV/STS to distribute food baskets to the vulnerable group within a week of linkage.<br><br>STS/TBHVs report to the TB cell weekly.
	Once the food basket is mobilised to the current PHI, the date of provision should be noted by TBHV in the co-designed "*Ni-Kshay Mitra* register".	This was done to document whether this prioritising mechanism works.
	Further, they should be prioritised for monthly nutritional support in a resource-constrained setting to facilitate receipt of a minimum of 6 baskets during their course of treatment.	
**Component 3**<br>Documentation of weight during the course of treatment	The documentation of weight gain in the TB treatment card was incomplete, based on the baseline OR findings.	Proxy indicator to determine the nutritional status post-prioritisation.

Supplementary Table 2: Monitoring indicators based on operational research findings from 2024-25 for addressing equity among vulnerable groups of adults with TB in Puducherry, India

		**Numerator**	**Denominator**
**1**	Consent for nutritional support	a) Adults with a BMI less than 18.5 who consented to nutritional support<br>b) a) Adults with drug-resistant TB who consented to nutritional support	All adults with TB notified
**2**	Early initiation of the first food basket among adults with TB	a) Number of adults with BMI less than 18.5 and BPL who received 1st food basket within 2 weeks<br>b) Number of adults with drug-resistant TB who received 1st food basket within 2 weeks	Adults with TB with a BMI less than 18.5 and BPL who consented to nutritional support<br><br>Adults with drug-resistant TB who consented to nutritional support
**3**	Number of food baskets distributed (overall)<br>a) Number of those with a BMI less than 18.5 who received six food baskets<br>b) Number of those with drug-resistant TB who received six food baskets		
**4**	Number of food baskets reserved for vulnerable groups of adults with drug-sensitive TB		

Supplementary Table 3. Socio-demographic and clinical characteristics of consented adults with TB consented for nutritional support between June and August 2025 (n=216) and 2023 (n=207), Puducherry

Characteristic	June-August 2023 (N=207)		June-August 2025 (N=216)	
**Individual characteristics**	**n**	**%**	**n**	**%**
Age (years) [Median (IQR)]	47 (39,59)		49 (38,60)	
**Gender**				
Male	142	68.6	146	67.6
Female	64	30.9	68	31.5
Transgender	1	0.5	2	0.9
**Residence**				
Rural	66	31.9	70	32.4
Urban	107	51.7	146	67.6
Not recorded	34	16.4	0	
**Occupation**				
Working	107	51.7	122	56.5
Not working	27	13	51	23.6
Not recorded	73	35.3	43	19.9
**Family-level Characteristics**				
**Socio-economic status**				
Below poverty line	143	80.8	196	90.7
Above poverty line	34	19.2	20	9.3
Not recorded	30	14.5		
Number of household contacts [Median (IQR)]	3(1,3)†		2(1,3)††	
**Behavioral Characteristics**				
**History of Tobacco use**				
No	169	81.6	173	80.1
Yes	30	14.5	38	17.6
Unknown	8	3.9	5	2.3
**History of alcohol use**				
No	149	72	150	69.4
Yes	51	24.6	60	27.8
Unknown	7	3.4	6	2.8
**Baseline BMI**				
More than 18.5	132*	64.4	141	65.3
Undernutrition (16-18.4)	47	22.9	41	19
Severe undernutrition (<16)	14	6.8	26	12
Very severe undernutrition (<14)	12	5.9	8	3.7
**Diagnosis and treatment details**				
**Site of Tuberculosis**				
Pulmonary	137**	66.2	146	67.6
Extra pulmonary	68	32.9	70	32.4
**Type of Tuberculosis**				
New	177	85.5	180	83.3
Retreatment	7	3.4	23	10.6
Drug resistant	23	11.1	13	6.1

Characteristic		June-August 2023 (N=207)		June-August 2025 (N=216)
**Classification of Tuberculosis**				
Bacteriologically confirmed	140	67.6	157	72
Clinically diagnosed	62	30	49	22.5
Not recorded	5.	2.4	12	5.5
**Drug combination taken**				
Fixed dose combinations	188	90.8	204	93.6
Loose drugs	18	8.7	12	5.5
Not recorded	1	0.5	2	0.9
**Comorbidity status**				
**HIV status**				
Non-reactive	203	98.1	206	95.4
Reactive	1	0.5	1	0.5
Unknown	3	1.4	9	4.1
**Diabetes status**				
Non-Diabetic	103	49.8	128	58.8
Diabetic	71	34.3	82	38
Unknown	33	14.9	7	3.2
**Treatment characteristics**				
**Treatment outcome**				
Died	11	5.3	10	4.6
Cured	106	51.2	130	60.2
Treatment complete	75	36.2	51	23.6
Treatment failure	4	1.9	1	0.5
Treatment extended	0		24	34
Loss to follow up	6	2.9	0	
Not recorded	4	1.9	0	

†7 data not recorded and †† 3 data not recorded

*2 data not recorded, **- 2(1%) PwTB in 2023 cohort had both pulmonary and EPTB manifestations
